# Rationally Designed Protein Building Blocks for Programmable Hierarchical Architectures

**DOI:** 10.3389/fchem.2020.587975

**Published:** 2020-10-29

**Authors:** Wenbo Zhang, Shanshan Mo, Mingwei Liu, Lei Liu, Lanlan Yu, Chenxuan Wang

**Affiliations:** ^1^State Key Laboratory of Medical Molecular Biology, Institute of Basic Medical Sciences, Department of Biophysics and Structural Biology, Chinese Academy of Medical Sciences and Peking Union Medical College, Beijing, China; ^2^Department of Chemistry, University of Wisconsin-Madison, Madison, WI, United States

**Keywords:** protein self-assembly, supramolecular nanostructures, protein-protein interactions, bioinspired materials, hierarchical construction

## Abstract

Diverse natural/artificial proteins have been used as building blocks to construct a variety of well-ordered nanoscale structures over the past couple of decades. Sophisticated protein self-assemblies have attracted great scientific interests due to their potential applications in disease diagnosis, illness treatment, biomechanics, bio-optics and bio-electronics, etc. This review outlines recent efforts directed to the creation of structurally defined protein assemblies including one-dimensional (1D) strings/rings/tubules, two-dimensional (2D) planar sheets and three-dimensional (3D) polyhedral scaffolds. We elucidate various innovative strategies for manipulating proteins to self-assemble into desired architectures. The emergent applications of protein assemblies as versatile platforms in medicine and material science with improved performances have also been discussed.

## Introduction

**Figure 1:**
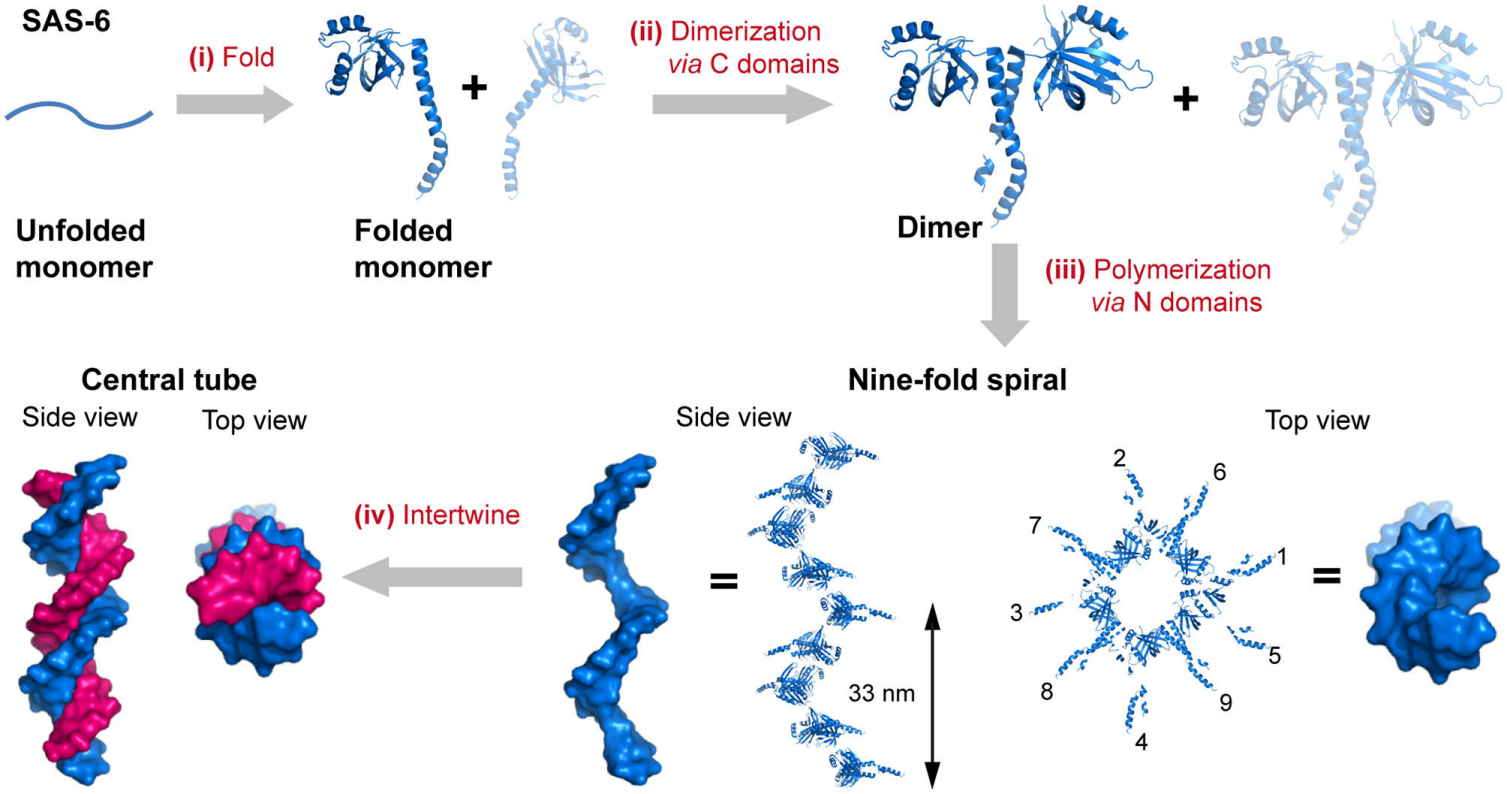
The hierarchical organization of SAS-6. (i) A linear SAS-6 polypeptide is folded into a tertiary structure composed of a C-terminal domain and an N-terminal domain. (ii) The dimerization of SAS-6 is achieved *via* a coiled coil formed by the C-terminal domains. (iii) SAS-6 dimers are polymerized into a 9-fold spiral *via* forming N-N dimerization interfaces. (iv) Two spirals intertwine around each other. SAS-6 structures are generated by using crystallography data (PDB ID 4GFC).

The self-assembly of proteins into an enormous range of molecular machines and structural scaffolds is one of the core principles for nature to create countless organisms, such as the oligomerization of protein kinases, transmembrane proteins, and signaling proteins, the polymerization of cytoskeletal proteins into intermediate filaments, and the emergence of membrane-less organelles induced by the phase separation of proteins (Nussinov et al., 2015; Boeynaems et al., 2018). As a matter of fact, nearly all of the structural proteins are identified to be polymers containing hundreds to millions of subunits, and most of the soluble proteins as well as membrane proteins are oligomers of two or more subunits (Harding and Hancock, 2008; Himanen et al., 2010; Nussinov et al., 2015). Three length scales of the hierarchy are frequently involved in the evolution of sophisticated biologically relevant hierarchical organization: the folding of a linear polypeptide into a well-defined secondary and tertiary structure, the nanocluster organization of proteins driven by intermolecular forces, and the arrangement of protein-nanoclusters into macroscopic superlattices. For example, the spindle assembly abnormal protein 6 (SAS-6), an essential component in the centrioles, is folded to be a central seven-stranded antiparallel open twisted β-sheet flanked by three α-helices (Hilbert et al., 2013). In particular, two helices and five β-strands of them are packed into an N-terminal globular domain, whereas a long α-helix and two β-strands constitute the C-terminal domain. Subsequently, the two C-terminal domain helices interlock with each other to render a coiled-coil bundle and lead to the dimerization of SAS-6. Multiple SAS-6 dimers further polymerize through the interactions across the interface between two N-terminal globular domains in proximity and form 9-fold symmetric ring-shaped spirals. Whereby the coiled-coil C-terminal domains are exposed at the outer surface and directed perpendicularly to the longitudinal axis of spiral. The length of the spiral is variable from 200 nm to several micrometers with an average screw pitch of 33 nm. Finally, two spirals intertwine around each other and build a 1D central tube of centrioles (Hilbert et al., 2013) (Figure 1). Not only limited to the case of SAS-6, but the stepwise hierarchical assembly throughout different length scales is also manifested in many naturally occurring proteins with versatile topological structures and broad functions. For example, the planar superlattices formed by bacterial surface layer proteins (S-layers) or purple membrane proteins in 2D, and the polyhedral lattice formed by clathrin or viral capsid in 3D (Ybe et al., 1998; Muller et al., 2006; Chung et al., 2010). Upon assembly, diverse protein nanostructures with distinct crystallographic point group symmetries can be constructed: (1) Cyclic groups. *C*_2_ symmetry (dimeric alcohol dehydrogenase), *C*_3_ symmetry (trimeric porin), *C*_4_ symmetry (tetrameric neuraminidase), and *C*_6_ symmetry (hexameric complement C1); (2) Dihedral groups. *D*_2_ symmetry (tetrameric phosphofructokinase), *D*_3_ symmetry (hexameric aspartate carbamoyltransferase), *D*_4_ symmetry (8-meric glycolate oxidase), and *D*_6_ symmetry (12-meric glutamine synthetase). (3) Cubic groups. *T* symmetry (12-meric protocatechuate 3,4-dioxygenase), *O* symmetry (24-meric ferritin), and *I* symmetry (60-meric satellite tobacco necrosis virus) (Goodsell and Olson, 2000).

The evolutionary selection of assembly endows proteins with a series of advantages in biological functionalities in five aspects: enhancing protein stability, building cooperativity, increasing local concentration, benefitting morphological function, and protecting signaling cascade. (1) Enhancing protein stability. Assembly reduces the protein surface area exposed to the solvent and enzymes, and thus improves the stability of protein against denaturation and proteolytic degradation (Goodsell and Olson, 2000; Nussinov et al., 2015; Mukherjee et al., 2018). For instance, insulin is produced and stored in granules as a hexamer. The hexamer is an inactive form of insulin and serves as a protecting architecture to prevent the formation of higher order amyloidal aggregates and the rapid degradation by enzymes. When insulin is released into the blood, the inactive hexamer dissociates into active monomers immediately and exert physiological functions (Mukherjee et al., 2018). (2) Building cooperativity between different protein subunits by clustering (Laskowski et al., 2009). Many allosteric enzymes are oligomeric clusters composed of two or more subunits that are arranged in a symmetrical way. When one subunit within the oligomer encounters a ligand, this subunit usually undergoes conformational changes which also affect the structural rearrangement of other adjacent subunits. As a consequence, the binding information propagates from one subunit to another and the coupling of motions among different protein subunits may either enhance or reduce the enzyme activity (Laskowski et al., 2009; Zhang et al., 2018). (3) Increasing local concentration and binding affinity of the functional proteins (Goodsell and Olson, 2000; Nussinov et al., 2015; Hnisz et al., 2017). Multiple independent binding sites are integrated into a multivalent active site which increases the binding strength of a ligand to the receptor. Once the ligand is trapped by one binding site, other spatially proximal binding sites make the dissociation of the ligand unfavorable. (4) Benefitting morphological function. The polymerization of structural protein is a feasible way to build sophisticated molecular machines in a variety of shapes with enough mechanical strength (Goodsell and Olson, 2000; Nussinov et al., 2015). Examples include the actin, a major constituent of the cellular cytoskeleton, which forms filaments and supports the shape of cell (Dominguez and Holmes, 2011); the polyhedral assembly of virus capsid proteins acting as a container to package either DNA or RNA (Wolf et al., 2010); and the ring-shaped connexon or perforin functioning as transmembrane channels (Liu et al., 1995; Yeager and Nicholson, 1996). (5) Protecting signaling cascade from the risk of signaling pathway promiscuity. For example, Ras is a kind of small GTPases and acts as a signaling switch in a number of molecular pathways. Half of Ras-family members are pre-organized as nanoclusters under normal physiological conditions. An interference with the association state of Ras can result in a diminished transport of signaling (Chen et al., 2018).

Revealing the evolution of sophisticated supramolecular architectures formed by the astronomical number of native protein assemblies has led to a profound understanding of the driving forces that govern the folding and assembly process of protein complexes. It motivates researchers to leverage this knowledge into the artificial design of novel protein folds for engendering predictable large-scale structures *via* a bottom-up approach. To date, the *ab initio* calculation to predict *de novo* protein folding and assembly from scratch is still challenging; the semi-empirical strategies that use the fragments from naturally occurring proteins as basic elements have been proved to be an efficient route to engineer the specific interactions in the biomimetic systems (Koga et al., 2012; Bungard et al., 2017). In this review, we aim to illustrate the nature-motivated design strategies of protein building blocks for constructing programmable assembled structures by introducing the recent progress made in this research field. Supramolecular protein assembly has become an emerging area at the interface between biomolecular science and nanotechnology, and represents an appealing opportunity in biotechnological and therapeutic applications. We also review and envision the application of artificially self-assembling protein architectures in materials science, biology, and medicine.

## Tertiary Structural Modules

α-Helix and β-sheet are the two basic secondary structure elements that can be exploited for the construction of building blocks in modular protein assembly. A specific packing of secondary structure elements forms tertiary structure and defines the size and geometry of individual protein modules. The spatial arrangement of side chains on the outside surface of tertiary building units provides non-covalent interactions to further assemble subunits with different tertiary structures into quaternary structures. In this section, we discuss the recent advances made to the *de novo* design of tertiary structural motifs by the rational packing of α-helices or β-strands.

**Figure 2:**
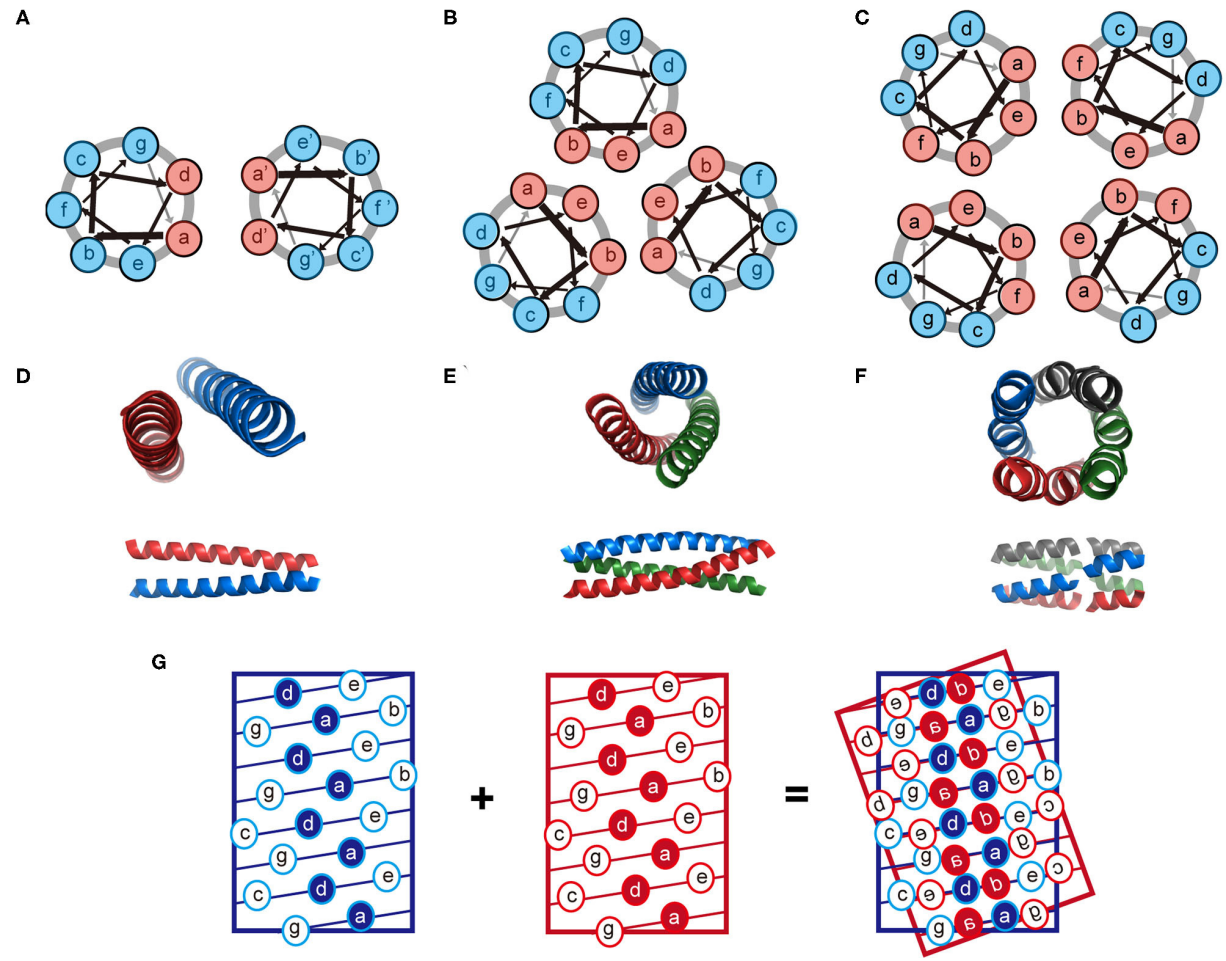
Schematic illustration of the coiled-coil structure. **(A–C)** Helix-wheel diagrams of the coiled-coil dimer **(A)**, trimer **(B)**, and tetramer **(C)**. Light red is referred to a non-polar residue and light blue is referred to a polar residue. Arrows with different thickness present the rotating manner of residues along the helix. (**D–F**) Coiled-coil dimer (**D**, PDB ID 4DZM), trimer (**E**, PDB ID 1OX3), and tetramer (**F**, PDB ID 2AG3) viewed from along the helix axes (top) and perpendicular to the helix axes (bottom). **(G)** A knobs-into-holes packing regime in a coiled-coil dimer.

The coiled-coil is a specific assembly of two or more α-helices that are aligned in either parallel or antiparallel fashion wrap around each other to form a supercoil (Woolfson, 2005; Woolfson et al., 2012). Coiled-coil motifs are one of the most abundant structural elements within protein tertiary structures and quaternary structures, estimated to be approximately 10% of the eukaryotic proteome (Knodler et al., 2011). A wide variety of coiled-coil architectures have been identified in native biological systems including: (1) dimer (tropomyosin), (2) trimer (fibritin and its mutants), (3) tetramer (the core domain of lactose repressor); (4) pentamer (cartilage oligomeric matrix protein), (5) hexamer (the core domain of the simian immunodeficiency virus gp41), and (6) larger oligomers (the bacterial membrane protein TolC containing 12 helices, the mechanosensitive channel MscS containing 21 helices, and cytotoxin ClyA containing 24 helices) (Bromley et al., 2008; Woolfson et al., 2012). The biological functions of coiled-coils can be summarized as protein-protein interlock domains (e.g., the intermediate filaments formed by keratin family and the microfilaments formed by actin) and DNA-binding domains (e.g., the leucine zipper domain of transcription factors, such as c-Fos, c-Jun, and GCN4) (Woolfson et al., 2012, 2015). The prevalence of coiled-coils in nature inspires extensive studies to reveal the molecular mechanisms underlying the oligomerization of helical bundles. Sequences that support a coiled-coil assembly feature an amphiphilic heptad repeat pattern denoted as [*abcdefg*]_n_. For a dimeric coiled-coil, the characteristics of heptad periodicity is [*HPPHPPP*]_n_, where *H* is referred to a hydrophobic residue and *P* is referred to a polar residue. Changes with the number and position of hydrophobic residues in repeated pattern lead to a different association state (Kohn and Hodges, 1998; Woolfson et al., 2012). Particularly, [*HHPPHPP*]_n_ occurs in trimeric coiled-coils; [*HHPPHHP*]_n_ occurs from tetramer to hexamer, and [*PHPHHPH*]_n_ anticipates above heptamer (Woolfson et al., 2012). Non-polar residues at positions *a* and *d* form a hydrophobic stripe along one side of the helix upon folding. The inter-helical attractions provided by the hydrophobic stripes drive different helices to pack together and lead to the formation of coiled-coils (Kohn and Hodges, 1998; Woolfson et al., 2012). The interaction interface of a coiled-coil is a characteristic knobs-into-holes packing regime dominated by hydrophobic side chains, in which a hydrophobic side chain from one helix interdigitates within a cluster of four hydrophobic side chains from the partner helix (Figure 2). The association state of a coiled-coil can be altered by a mutation with the residues at positions *a* and *d*. For instance, the oligomerization state for GCN4-pIL is dimer where *a* is isoleucine and *d* is leucine. Then the dimer transforms into a trimer for GCN4-pII (*a* = *d* = isoleucine), and tetramer for GCN4-pLI (*a* = leucine, *d* = isoleucine) (Kohn and Hodges, 1998; Woolfson et al., 2012). The hydrophobic core of a coiled-coil is flanked by the polar residues at positions *e* and *g*, which are highly conserved charged residues. The electrostatic interactions between helical partners can be used to toggle the combinations of helices between homo- and hetero-arrangement (Vo-Dinh, 2005). For example, tropomyosin derived peptide with a heptad repeated sequence of LEALEGK presents glutamic acid and lysine at positions *e* and *g*, respectively, and thus the *e*_*A*_-*g*_*B*_ and *e*_*B*_-*g*_*A*_ inter-helical electrostatic attractions between two interacting helices, *A* and *B*, fold peptide into a homodimeric conformation (Vasquez et al., 1987). Similarly, electrostatic interactions drive GCN4 inspired peptides Basic-p1 (carrying cationic flanking sides) and Acid-p1 (carrying anionic flanking sides) to co-assemble into a heterodimeric coiled-coil and prevent the formation of homodimer (Arndt et al., 2000).

**Figure 3:**
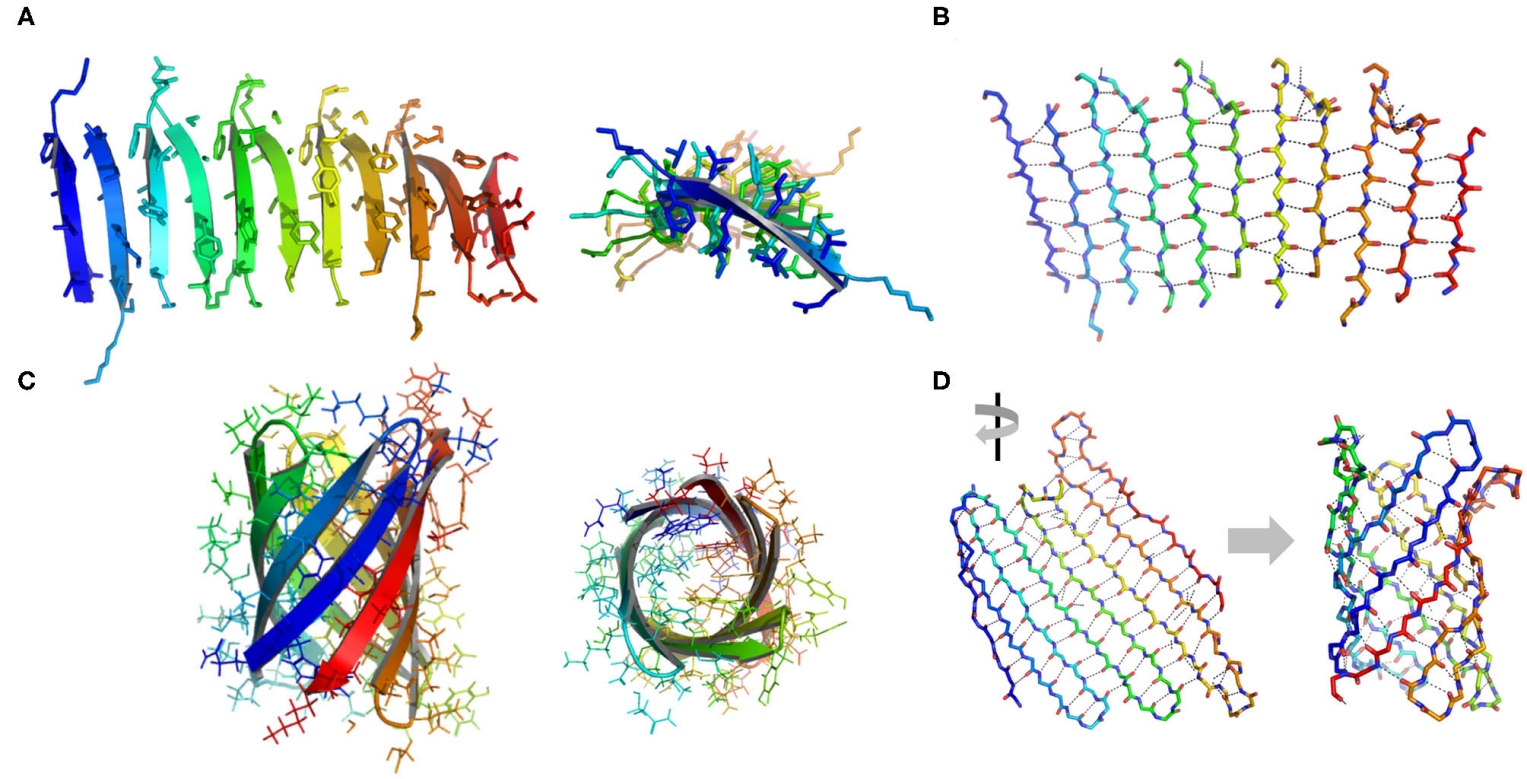
Illustration of β structures. **(A)** A representative β-sheet structure (PDB ID 5YS7) viewed from perpendicular to the cross-β fibril (left) and along the growth direction of a cross-β fibril (right). **(B)** The hydrogen bond arrangement in a cross-β fibril. **(C)** A representative β-barrel structure (PDB ID 1JMX). Left: side view. Right: top view. **(D)** A 2D extended β sheet (left) rolls up to form a 3D β barrel (right). Hydrogen bonds are indicated by dash lines.

β-Sheets are another important type of structural motifs that are involved in protein association (Figure 3). There are multiple recorded β-sheet rich protein architectures in nature, such as cross-β fibrils (amyloidal proteins and their core segments), β-barrels (porins, pre-proteins translocases, and lipocalins), β-annulus (β-annulus peptides from tomato bushy stunt virus capsids), and β-propellers (the influenza virus protein viral neuraminidase, β-transducin repeat, and β-propeller phytases) (Luo et al., 2016; Pieters et al., 2016). Different from a coiled-coil in which side chains dominate the packing of secondary units, a β-sheet presents extensive hydrogen bonds between protein backbone as well as side-to-side interactions. Taking the assembly of cross-β structure as an example: the non-polar side chains within an amphiphilic linear peptide (e.g., native amyloidal protein A and its core segment KLVFF, and *de novo* designed peptides RADA16-I and EAK16-II) engage in hydrophobic interactions and drive peptides to aggregate (Zhang and Rich, 1997; Yokoi et al., 2005; Pizzi et al., 2017). The inter-peptide hydrogen bonds between the amide moieties from adjacent peptides exclude interfacial water from the proximity of peptide backbones. As a consequence, the amphiphilic peptide adopts a cross-β fibril conformation where individual β-strands are continuously stacked with each other. The growth direction of cross-β fibril is perpendicular to the individual β-strands. Ideally, the extension of cross-β fibril is infinite. An issue emerging from the study of cross-β is such structural element lacks an efficient limit switch that sets a boundary for the polymerization of β-sheets (Mao et al., 2011; Wang et al., 2012). Toward the generation of well-defined tertiary architectures with finite volume by exclusive β-sheets, attempts have been made to artificially create mimetics of other types of β-rich protein assembly modules. However, the *de novo* design of such a β structure, e.g., β-barrel and β-propeller, is an outstanding challenge due to the tendency of all-β proteins to associate and form amyloidal aggregates (Terada et al., 2017; Dou et al., 2018). For example, a β-barrel is a tandem repeated structure that β-strands are twisted around a central axis to form a closed structure in which the first strand is bound to the last strand via hydrogen bonds (Dou et al., 2018). Each β-strand is staggered onto the adjacent one, leading to an inter-strand registry shift. Two key structural elements impair the stability of a β-barrel. First, the steric repulsions between side chains parallel to inter-strand hydrogen bonds break the strand pairing of β-sheet strands. Second, the chirality of some specific residues is not favorable to the twist of β-strands within a β-barrel. Thus, the rational β-barrel design requires a careful placement of specific residues at each position (Dou et al., 2018). There are a small number of artificial β-sheet rich proteins from scratch in document, such as a *de novo* fluorescence-activating β-barrel, a fully symmetric β-propeller predicted from the 1RWL template, and a complex α/β protein involving strand-strand, strand-helix, and helix-helix interfaces (Koga et al., 2012; Terada et al., 2017; Dou et al., 2018).

## One-Dimensional Assembly Structures

Architectures formed by the addition of units in one direction are defined as 1D structures. Typically, 1D assembly architectures include (1) String. Protein units are connected in a head-tail fashion; (2) Closed-ring. A string that is bent and coupled end-to-end to form a closed circle; (3) Tubule. A linear structure with a ring-like cross-section perpendicular to the growth direction (Sun et al., 2017). The construction of 1D protein nanostructures can be achieved via a delicate design of protein-protein interactions to control the orientation of assembled proteins. Representative strategies and practical examples will be introduced as follows.

### Protein Strings

**Figure 4:**
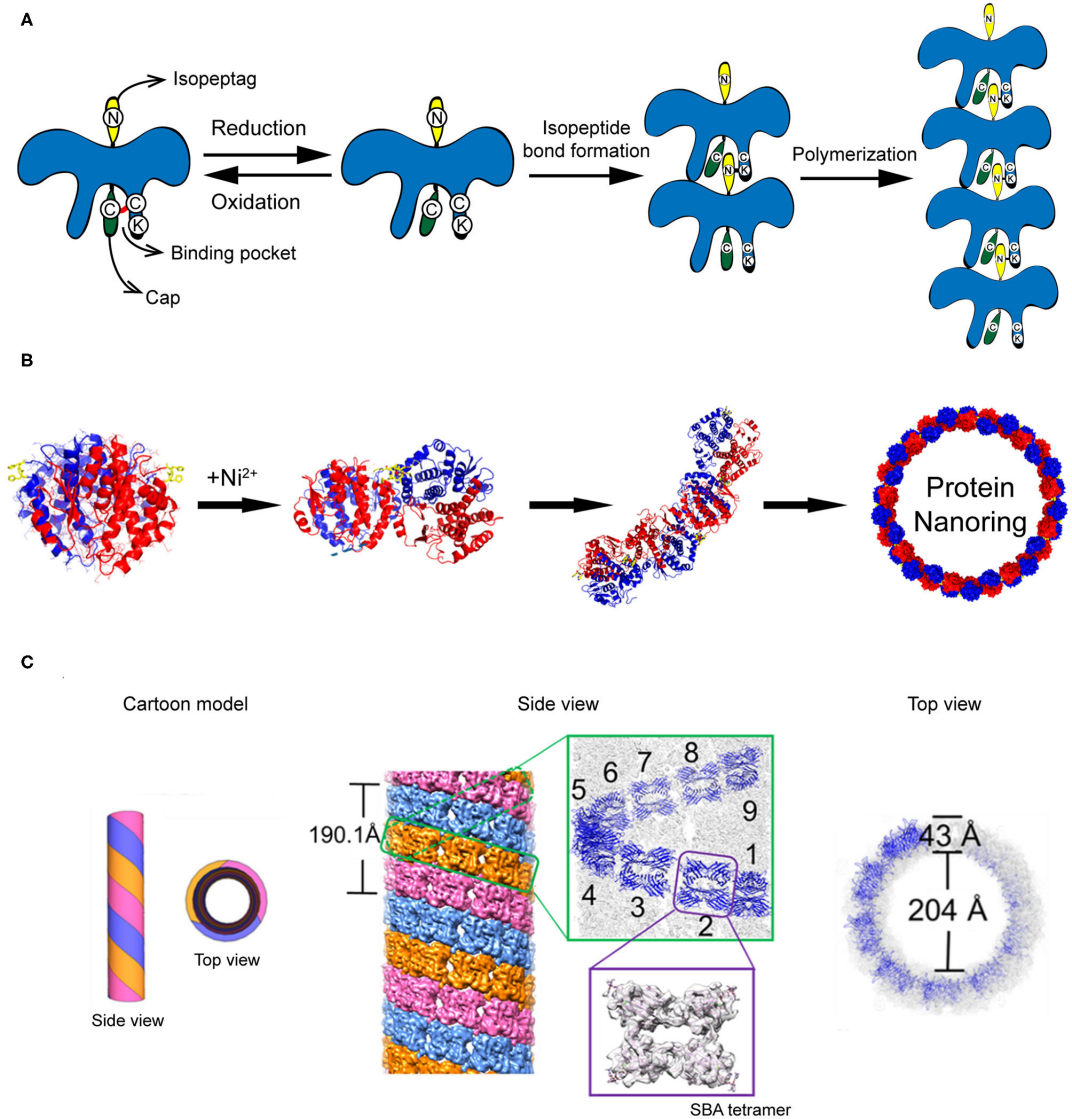
Self-assembled 1D protein structures. **(A)** The nanochain formation based on the assembly of recombinant PS proteins. **(B)** The nanoring formed by Ni^2+^-His coordination. Reproduced with permission from Bai et al. (2013). Copyright 2013 American Chemical Society. **(C)** The formation of microtubule structure driven by glycoprotein recognition and Rhodamine B dimerization. Reproduced with permission from Yang et al. (2016). Copyright 2016 American Chemical Society.

The construction of protein strings can be directed by diverse strategies including computational design, fusion protein, and chemical cross-linking strategies. (1) Computational design approach. Shen and co-workers reported a building of helical protein filaments by using Rosetta combinatorial sequence optimization (Shen et al., 2018). Fifteen *de novo*-designed helix repeated proteins (DHRs) with desirable protein-protein interaction interfaces were constructed and polymerized into micrometer-scale filaments with different molecular geometries. The diameter of filaments can be regulated by changing the repeat unit number of individual DHR building blocks. The growth of DHR-assembled filament is dynamically controlled by adding capping units to terminate the polymerization. (2) Fusion protein approach. Yeates et al. genetically fused two distinct proteins, i.e., carboxylesterase and influenza virus matrix protein M1, to generate a Janus protein building block (Padilla et al., 2001). The free monomers of either M1 or carboxylesterase can spontaneously cluster into homogeneous oligomers. After fusion, the M1-carboxylesterase Janus protein retains the oligomerization ability encoded by M1 and carboxylesterase and aggregates into long filaments with 4 nm in width and over 200 nm in length. (3) Chemical cross-linking approach. Isopeptag is a 16 amino acid peptide tag identified from the split *Streptococcus pyogenes* pilus subunit (Spy0128) (Zakeri and Howarth, 2010). The binding of isopeptag to its target receptor results in a spontaneous formation of an irreversible amide bond between an asparagine side chain of isopeptag and a lysine side chain of the receptor, making the interactions between isopeptag and its receptor significantly stable even under extreme conditions (Zakeri and Howarth, 2010). Inspired by the structure of Spy0128, Matsunaga et al. (2013) constructed a recombinant protein building block PS, in which an isopeptag is attached to the N-terminus of PS and an isopeptag binding pocket is presented in the C-terminal domain. Under oxidative conditions, a cap peptide ligand, an isopeptag mimic but inert to form intermolecular amide bond, is stuck in the C-terminal binding pocket via a disulfide bond and prevents the docking of isopeptag into the binding pocket. Under reductive conditions, the disulfide bond between cap peptide and the pocket is cleaved. The N-terminal isopeptag is allowed to insert into the binding pocket and form an amide bond between asparagine and lysine side chains. Finally, multiple monomers are linearly polymerized in a head-to-tail fashion (Figure 4A). The initiation and termination of the building blocks assembly can be switched by the redox condition of solution.

### Protein Rings

In addition to stringing together monomers to form a linear chain, proteins can also be arranged to form a ring-like structure by carefully manipulating the protein-protein interaction interface. One approach to associate protein units into nanoring is to manipulate the non-covalent interactions across the protein-protein interface. Bai et al. (2013) used a natural homodimer sjGST-2His (a variant of glutathione S-transferase from *Schistosoma japonicum*) as a skeleton module and conjugated different sjGST-2His dimers via metal-coordination interactions. The sjGST-2His contains two properly oriented His metal-chelating sites (His137 and His138) on the protein surface and the two subunits are related by a 2-fold axis (*C*_2_ symmetry). When a chelating metal ion Ni^2+^ is introduced into the solution at low concentration, the Ni^2+^-His coordination interactions drive the growth of the protein assemblies in a fixed bending direction and finally lead to the formation of nanoring (Figure 4B). In principle, the magnitude of metal-coordination interactions is negatively correlated to the ionic strength of the solution, which is described by the Debye-Hückel equation. Thus, one would predict that the geometry of nanoring can be modulated by changing the ionic strength of the solution. In fact, an increase in the curvature of sjGST-2His/Ni^2+^ nanoring was observed as the ionic strength of the solution increased.

### Protein Tubules

Compared to the protein string and protein ring described above, a more complicated protein-protein interaction design is required to achieve protein tubules. A tubular structure needs to be built up with a ring-like structure in the horizontal direction and a linear growth in the vertical direction. For example, protofilament spirally coiled microtubule is a significant self-assembled architecture found in nature with crucial biological function, such as one of the key components of the cellular cytoskeleton. To mimic the protein microtubule structures existing in nature, artificial supramolecular microtubules were designed by using two types of intermolecular interactions: protein-saccharide recognition and π-π stacking interaction (Yang et al., 2016). Planar tetrameric soybean agglutinin (SBA) protein is selected as a skeleton element for building the microtubule structure. To cluster different SBA tetramers, a small organic ligand composed of a saccharide moiety of either *N*-acetyl-α-_D_-galactosamine (GalNAc) or α-_D_-galactosamine (Gal) and a Rhodamine B moiety is synthesized. The saccharide moiety anchors an SBA tetramer via protein-saccharide interactions, whereas the π-electron-rich Rhodamine B moiety drives two small organic ligands to stack with each other. As a consequence, SBA and the small organic ligand co-assemble into protofilaments. Three protofilaments further twist around each other and form a microtubule (Figure 4C). In the horizontal direction, the tubular structure features a central hole of 20 nm in diameter with a wall of 4 nm in thickness. In the vertical direction, three protofilaments wind together in a left-handed helical fashion with a period of ~19 nm, which is corresponding to 9 SBA tetramers. This artificial supramolecular protein microtubule shows comparable structures to the native protofilament spirally coiled microtubule.

## Two-Dimensional Assembly Structures

To date, a variety of 2D protein arrays have been ingeniously constructed. Different from the building of 1D assembly structures, where protein-protein interactions in one direction are usually involved, protein-protein interactions from different directions are needed to achieve the more complicated 2D architectures. This part will emphatically introduce the typical 2D protein-assembled architectures and the relevant strategies for manipulating protein-protein interactions, including computational design, symmetry-guided assembly, metal-ligand coordination driven assembly, and template-directed assembly.

### Computational Design Guided Protein Assembly

**Figure 5:**
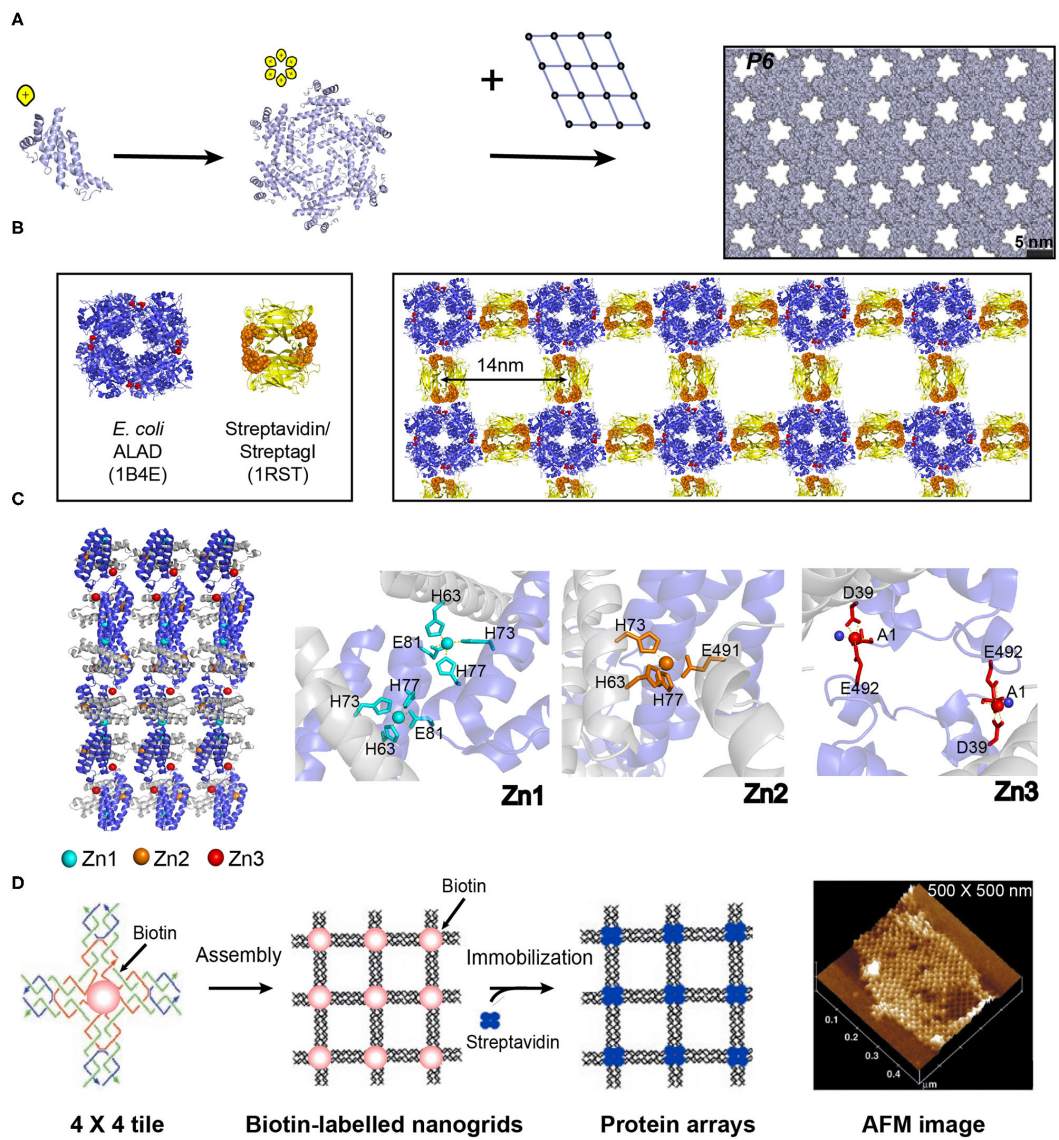
Self-assembled 2D protein arrays generated by different strategies. **(A)** The protein-based 2D lattice (*P*6) generated by the computational design strategy. Reproduced with permission from Gonen et al. (2015). Copyright 2015 The American Association for the Advancement of Science. **(B)** The 2D crysalin lattice generated by the symmetry-guided protein assembly strategy. **(C)** The molecular arrangement in a 2D Zn^2+^-RIDC3 sheets (Left). Three types of Zn^2+^ coordination interactions that enable the self-assembly of RIDC3 (Right). **(D)** 2D arrays generated by the template-directed strategy using 4 × 4 DNA nanogrids via biotin-streptavidin interactions. Adapted with permission from Yan et al. (2003). Copyright 2003 The American Association for the Advancement of Science.

Numerous theoretical simulation methods, such as Z-Dock, Rosetta, Amber, and GRAMM-X, have been developed to guide the *de novo* design of the interfacial properties and recognition behaviors of protein building blocks with atomic resolution (Kortemme and Baker, 2004; Rodrigues and Bonvin, 2014). The key idea of these sampling algorithms is to search for the conformational space of proteins and scoring functions to deduce the possible binding affinities. Based on the quality of the energy function and the adequate sampling of conformations, shape-complementary protein-protein interfaces with low-energy are generated by these programs (Vajda and Kozakov, 2009; Moal et al., 2013). For instance, Baker and co-workers described a *de novo* approach to design large planar 2D protein arrays using Rosetta (Gonen et al., 2015). In principle, there are totally 17 distinct plane groups of a protein unit cell to pack into a periodic 2D layer, including the oblique crystal system (*p*1, *p*2), rectangular crystal system (*pm, pg, cm, p*2*mm, p*2*mg, p*2*gg, c*2*mm*), square crystal system (*p*4, *p*4*mm, p*4*gm*), and hexagonal crystal system (*p*3, *p*3*m*1, *p*31*m, p*6, *p*6*mm*) (Zou et al., 2011). To reduce the complexity in computational simulation, the plane groups involving the minimum number of the interfaces with the adjacent unit cells were selected and aligned by the cyclic protein homo-oligomeric structures documented in the Protein Data Bank (PDB). The optimization with the degrees of freedom and sequence design calculation were carried out to identify the low-interaction-energy conformations with shape-complementary interfaces. Finally, the protein building blocks that can self-assemble into a planar lattice with a plane group of *P*321, *P*42_1_2, or *P*6 were created (Figure 5A). Other types of *de novo* 2D symmetrical architectures were reported by the Tame and Bradley groups, in which a 6-fold symmetrical β-propeller protein and α-helical tandem repeat proteins with closed architectures were designed by Rosetta-based software packages (Voet et al., 2014; Doyle et al., 2015). The formation of 2D plane structures by the predicted protein units was validated by X-ray crystallography. Work from these groups demonstrates the feasibility of *de novo* computational approaches to create 2D supramolecular patterns.

### Symmetry-guided Protein Assembly

Symmetry is one of the most important selection roles by which nature creates versatile architectures. At the atomic level, symmetrical assembly provides an important way for atomic cluster to reach the lowest energy state (Hoare and Pal, 1975; Goodsell and Olson, 2000). For example, in the nucleation of rare-gas atoms, where unidirectional van der Waals forces dominate the interactions between atoms, the high-symmetrical packing manner is energetically favorable (Hoare and Pal, 1975). At the more complicated biomolecular levels, there is also a bias toward symmetrical structures. For instance, the native supramolecular systems that were made by the assembly of the same subunits always arrange subunits in a symmetrical pattern (Crick and Watson, 1956; Andre et al., 2008; Lai et al., 2012). Indeed, the symmetrical homo-oligomers are widely prevalent, constituting 50–70% of all proteins with a quaternary state (Levy et al., 2008). In analogy to the energy-driven atomic homo-oligomerization, the prevalence of symmetrically homo-oligomeric proteins is proposed to stem from the evolutionary stress that the interfacial interactions between subunits favor the choice of symmetric arrangement (Cornish-Bowden and Koshland, 1971; Goodsell and Olson, 2000).

The numerous homo-oligomers in nature provide valuable insights on structural elements for investigators to design 2D non-covalent architectures based on the combination of symmetrically self-associating interfaces (Luo et al., 2016). For example, Sinclair et al. (2011) chose the structurally defined proteins streptavidin and aminolevulinic acid dehydrogenase (ALAD) as building blocks. Streptavidin is a *D*_2_-symmetric tetramer with one streptagI peptide binding site per subunit. ALAD is a homo-octamer with *D*_4_-symmetry. When the *D*_4_-symmetric ALAD octamer is fused with a streptagI peptide at the each of the eight C-termini, the streptagI peptide acts as a linker to fasten an ALAD with a streptavidin leading to the generation of a fusion protein, crysalin. Under physiological conditions, crysalin self-assembles into a 2D lattice with a *P*422 symmetry (Figure 5B) (Sinclair et al., 2011).

### Metal-Coordination-Driven Protein Assembly

In addition to the interface interactions between subunits, metal ions in protein solutions can have coordination interactions with monomeric proteins and facilitate the assembly of proteins. For example, a monomeric protein-cytochrome 3 (RIDC3) that carries Zn^2+^ coordinating motifs (two metal-chelating bis-histidine motifs: His59/His63 and His73/His77) forms 2D crystalline arrays induced by the interactions with Zn^2+^ (Figure 5C) (Brodin et al., 2012). The addition of 300 μM Zn^2+^ into the solution containing of 100 μM RIDC3 at pH 6.5 triggered the onset of Zn^2+^-RIDC3 hierarchical organization, where three steps are involved. First, a Zn^2+^ (named as Zn1) binds to the His73 and His77 residues from one RIDC3 monomer and the His63 residue from another monomer, leading to a *C*_2_-symmetric dimerization as the initial step of Zn^2+^-RIDC3 complex nucleation. Second, the Zn^2+^-protein intermolecular interactions drive the packing of RIDC3 dimers along two orthogonal directions. Along the vertical axis, two *C*_2_-dimers are interconnected into a tetramer through the coordination interactions between Zn1 and Glu81. Along the horizontal axis, multiple tetramers are interlocked by Zn^2+^ ions (represented as Zn2) in a head-to-tail configuration, generating a 1D supramolecular chain which is connected by pairwise Zn^2+^-Glu49 interactions. The 1D supramolecular chain presents unoccupied Zn^2+^ coordinating sites (Asp39 and Glu49) on the sides. Finally, additional Zn^2+^ ion (labeled as Zn3) binds to Asp39 and Glu49, packing different 1D chains in a parallel manner into an ordered 2D crystalline sheet (length ~5 μm and width ~1 μm). This 2D assemblies exhibited long-term stability over 6 months at room temperature and maintained intact after a dilution into the solution without Zn^2+^.

### Template-Directed Protein Assembly

The above mentioned three strategies for designing ordered 2D protein assemblies require the protein building blocks bearing self-associating interfaces to interact with each other. Thus, a different methodology is needed to achieve an ordered arrangement for the proteins that are unable to self-assemble. To address this challenge, an alternative approach is suggested: using a 2D pre-organized periodic structure as a host scaffold to template the adsorption of guest proteins. A diverse range of lattice-like supramolecular systems are available for guiding the nanopatterning of protein, such as DNA origami and naturally occurring protein lattice (Niemeyer et al., 1994). A key step in the development of host template is the design of an intermolecular recognition motif with a strong and specific binding capability to immobilize proteins and substantial efforts have been made in this field. (1) Specific intermolecular interactions are used to organize the guest proteins. For instance, a 4 × 4 DNA tile with four four-arm DNA junctions pointing in four directions and one biotin group at the tile center was designed. The 4 × 4 tiles associate with each other into an ordered 2D nanogrid with a size of 500 × 500 nm. The active biotin groups presenting on the nanogrid trap streptavidin proteins from solution to generate a periodic 2D streptavidin layer (Figure 5D) (Yan et al., 2003). (2) The guest proteins are covalently immobilized on the template. For example, a series of guest proteins, such as streptavidin, fluorescent proteins, IgG-binding domain, and methyl parathion hydrolase can be genetically fused with an S-layer protein. S-layer protein, a major component of bacterial cell surface, displays a strong potential to assemble into a monolayer. The self-assembly of S-layer protein renders the form of a 2D crystalline layer and regularly spaces the guest proteins on the surface of the S-layer superlattice in defined repetitive spacing (Moll et al., 2002).

## Three-Dimensional Assembly Structures

Relative to the 2D planar sheets described above, the construction of 3D protein-based hierarchical architectures, such as vesicles, cages, crystal frameworks, and hydrogels, etc., are more complicated and challenging. Till now, the 3D supramolecular structures have demonstrated a wide and fascinating application in the fields of nanoscience, material science, and synthetic biology.

### Computational Design Guided Protein Assembly

**Figure 6:**
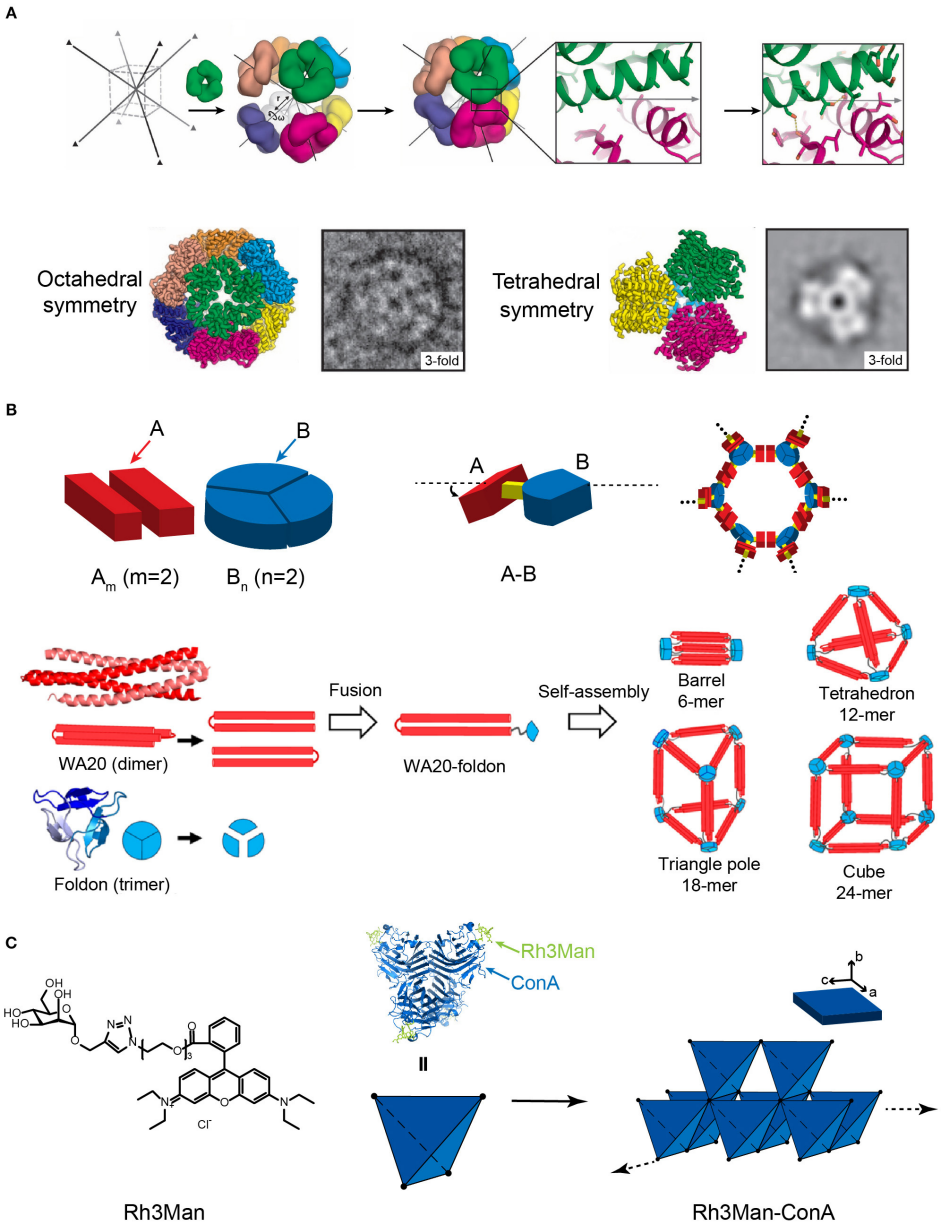
Self-assembled 3D protein superstructures generated by different strategies. **(A)** Cagelike protein assemblies with octahedral and tetrahedral point group symmetries generated by the computational strategy. Adapted with permission from King et al. (2012). Copyright 2012 The American Association for the Advancement of Science. **(B)** 3D architectures generated by the symmetry-matching fusion protein assembly strategy. The general strategy for designing fusion proteins that assemble into symmetric nanostructures (top). The 3D supramolecular structures constructed by WA20 and Foldon (bottom). Reproduced with permission from Kobayashi et al. (2015). Copyright 2015 American Chemical Society. **(C)** 3D crystal generated by the ligand directed protein assembly strategy based on the co-assembly of ConA and Rh3Man.

Various types of cage-like protein nanomaterials were *de novo* designed by Baker lab to mimic the virus capsid structures. Symmetric protein oligomers were selected from PDB as building blocks and aligned with a space group to reach a low-energy protein-protein interface between oligomers. In the first case, eight *C*_3_-symmetric protein trimers were chosen as building blocks, symmetrically located in each vertices of a body-centered cubic space lattice with directions along center-to-vertex. The energy of protein trimers was subsequently optimized by RosettaDesign calculation to achieve a shape complementarity in the system with a minimum number of buried unsatisfied hydrogen bonds at the interfaces between different packing units. To validate the design methodology experimentally, the assembly of *C*_3_-symmetric protein trimers, characterized by Cryo-EM, displays two sets of supramolecular structures, i.e., an expected product (24-subunit nanocage with octahedral symmetry) and a side reaction product (12-subunit cage with a tetrahedral symmetry) (Figure 6A) (King et al., 2012). To increase the accuracy of *de novo* prediction, an alternative strategy was proposed that use two types of distinct proteins to construct a two-component protein nanocage (King et al., 2014). For example, a 120-subunit icosahedral nanostructure with an apparent molecular weight of 1.8–2.8 mega Dalton has been created by the co-assembly of two types of subunits (Bale et al., 2016). The icosahedron exhibits a large cavity volume with a diameter of 24–40 nm, which enables a controllable package of macromolecular cargoes, such as green fluorescent proteins. This achievement indicates that the symmetric modeling combined with the computational design of protein-protein interface injects new excitement into the field of designing large protein architectures with high accuracy.

### Symmetry-Matching Fusion Protein Assembly

Yeates and coworkers demonstrated a proof of concept using the symmetry-matching fusion strategy to construct a wide range of assembly nanoscale protein architectures. One subunit of protein *A*, which oligomerizes into a symmetric *A*_*m*_, is genetically fused by a rigid linker to another subunit of protein *B*, which oligomerizes into a symmetric *B*_*n*_(*m* and *n* represent the number of units). The fusion protein, *A-B*, retains the association ability encoded by the *A* and *B* oligomerization domains and forms an ordered lattice (Figure 6B). To control the curvature of assemblies, a rigid linker is introduced to connect the two structural domains and fix the symmetry axes of two domains intersecting at a particular angle. For example, a dimeric M1 matrix protein with a 2-fold rotational symmetry and a trimeric biomoperoxidase with a 3-fold rotational symmetry were genetically fused by a short intervening α-helical linker in a pre-determined orientation (Padilla et al., 2001). The fused protein M1 matrix-biomoperoxidase self-associates into a 12-subunit tetrahedral cage. Another representative example is the fusion protein of cowpea chlorotic mottle virus capsid protein (CP) and elastin-like polypeptide (ELP). CP is an interesting building block that can be packed into a spherical shell in icosahedral symmetry involving 2-, 5-, and 6-fold rotation axes. Triangulation number (*T*) is defined as the square of the distance between two adjacent 5-fold vertices, which is used to describe the topology of the protein shell. At low pH and room temperature (pH 5, 20°C), the self-assembly of CP units drives the fusion proteins CP-ELP associate into a *T*-3 nanocapsule with a diameter of 28 nm. This nanocapsule presents an outer shell formed by pentameric CP domains and an inner shell composing of ELP domains. Increased pH (pH 7.5, 20°C) weakens the interactions among CPs and dissociates the *T*-3 nanocapsule into CP-ELP dimers. High temperature (pH 7.5, 37°C) triggers the thermally responsive aggregation mediated by the ELP domains. The assembly of ELPs converts the association state of CP-ELP into a *T*-1 particle with a diameter of 18 nm (Van Eldijk et al., 2012). Besides cage-like architectures, diverse 3D morphologies have been successfully prepared by applying the symmetry-matching fusion strategy. Kobayashi et al. created a 4-fold helical bundle by a dimeric α-helical hairpin protein WA20. One bundle end is capped by a three-arm branched junction composing of β-propeller-like trimeric protein, Foldon (Kobayashi et al., 2015). The self-assembly of fusion protein WA20-Foldon gives rise to the formation of a set of architectures, including the barrel-like hexamer, the tetrahedron-like 12-mer, triangle-pole-like 18-mer, and cube-like 24-mer protein assemblies (Figure 6B). More efforts were subsequently made toward the generation of cube-shaped, and porous nanostructures by using symmetry-matching fusion protein. 2-Keto-3-deoxy-6-phosphogalactonate (KDPGal) aldolase and the dimeric domain of FkpA are carefully connected by a four-residue linker to fix the symmetry axes of KDPGal and FkpA domains intersecting at an angle of 35.3°. The KDPGal-FkpA fusion protein self-assembles into an octahedral symmetric cubic cage (750 kDa) with an outer diameter of 225 Å and an inner cavity of 130 Å in diameter (Lai et al., 2014).

### Ligand Directed Protein Assembly

Specific ligands that facilitate protein-protein interactions can be used as a molecular partner to co-assemble with proteins. For the situation of small organic molecules, Rhodamine is often used as a skeleton element and provides the driving forces for inducing protein assembly due to the π-π stacking interactions between different Rhodamine moieties. For example, a heterozygous molecule Rh3Man was synthesized to contain a Rhodamine domain to induce the dimerization of Rh3Man, and a saccharide domain to specifically recognize a native protein concanavalin A (ConA) and form an Rh3Man-ConA complex. As a result, the two types of non-covalent interactions, the π-π stacking between Rhodamine and the saccharide-ConA recognition, lead to the formation of a large-sized 3D framework (ca. 200 μm × 100 μm × 20 μm) (Figure 6C) (Sakai et al., 2014). An alternative approach to build a 3D architecture is introducing additional interactions in the vertical directions of the pre-existing 2D planar sheets (i.e., layer-by-layer stacking). For example, the immobilization of Rhodamine Red C2 maleimide on the surface of Zn^2+^ mediated 2D RIDC3 planar sheets can align multiple 2D RIDC3 layers perpendicularly into a 3D crystal via Zn^2+^ coordination interactions (Brodin et al., 2014).

### The Assembly of Amphiphilic Protein

**Figure 7:**
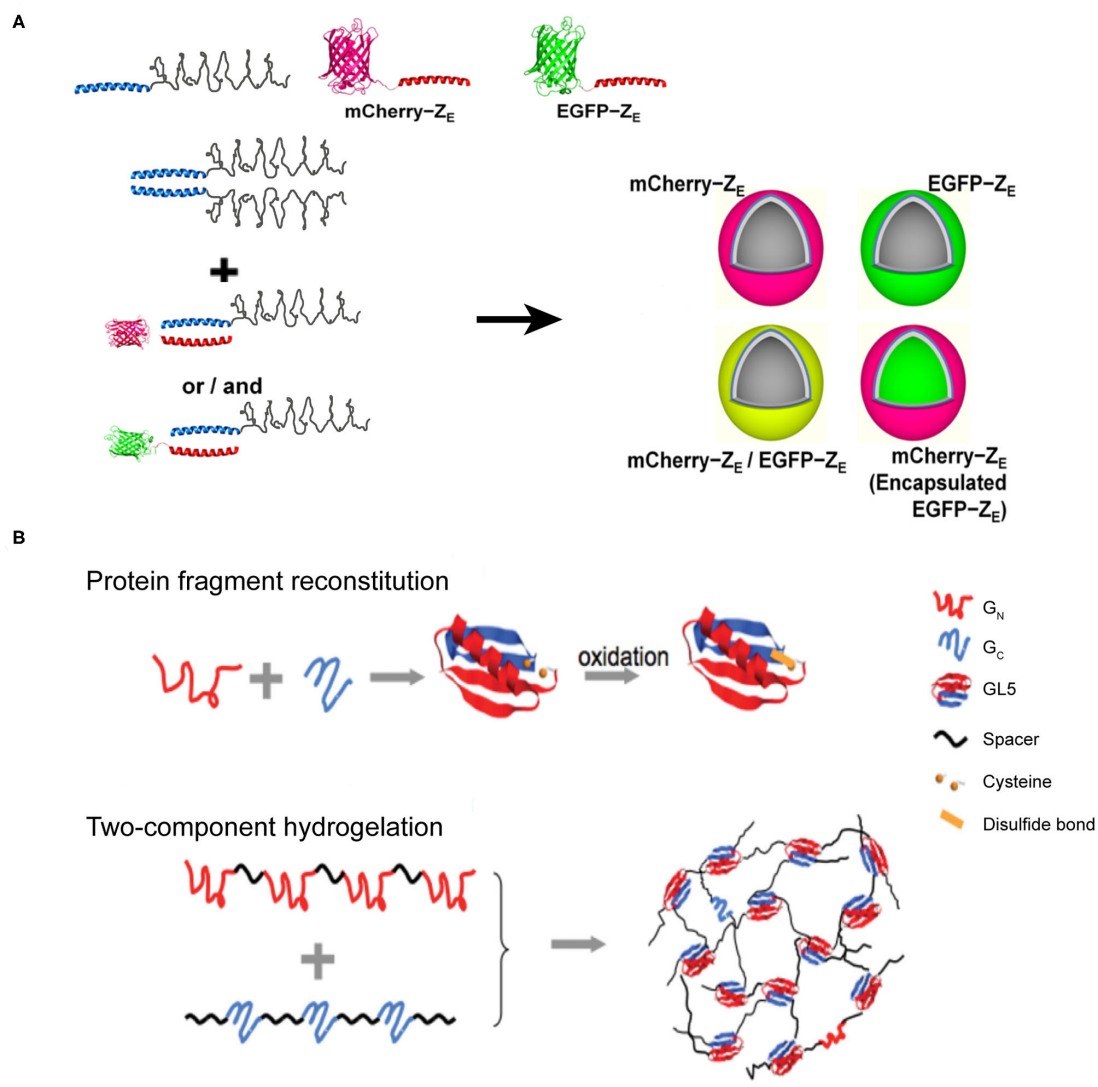
Self-assembled 3D protein superstuctures generated by amphiphilic protein and protein fragment complementation directed protein assembly. **(A)** Recombinant protein amphiphiles and their self-assembly into vesicles. Reproduced with permission from Park and Champion (2014). Copyright 2014 American Chemical Society. **(B)** Formation of reconstituted GL5 from GN and GC (Top) and the schematic of fragment complementation induced two-component hydrogelation. Reproduced with permission from Kong and Li (2015). Copyright 2015 Wliey-VCH.

Amphiphilic molecules are widely used in constructing nano-assemblies that is in fact beyond the scope of this section. Herein, we only address a typical protein-assembly process mediated by coiled-coil motifs, involving the hydrophilic and hydrophobic interactions. An arginine-rich leucine zipper motif (Z_R_) was genetically fused with a hydrophobic ELP to achieve an amphiphilic fusion protein, Z_R_-ELP (Park and Champion, 2014) (Figure 7A). Whereas, the hydrophobic ELP domain provides the driving forces for the micellization of Z_R_-ELP. Analogous to the arginine-rich leucine zipper motif, a glutamic acid-rich leucine zipper motif (Z_E_) was conjugated to a hydrophilic fluorescent protein, either mCherry or EGFP, to generate two hydrophilic fusion proteins, mCherry-Z_E_ and EGFP-Z_E_. The strong heterodimerization potency between Z_E_ and Z_R_ domains to form a leucine zipper coiled-coil induces Z_R_-ELP to co-assemble with either mCherry-Z_E_ or EGFP-Z_E_ into hollow vesicles with a diameter up to 1 μm. The inner vast space of such protein vesicles hints on the application of this supramolecular system as a drug delivery system to encapsulate various types of cargoes.

### Protein Fragment Complementation Directed Protein Assembly

Protein-fragment complementation, also referred as protein fragment reconstitution, is a protein folding mechanism by which protein fragments can reconstitute the folded conformation of the native protein when split into two complementary parts. The reconstitution process of two complementary protein fragments into a native-like conformation provides a driving force for co-assembling protein-based supramolecular system (Kong and Li, 2015). For example, a split protein fragment *A* is hybridized with a spacer unit *S* to generate an artificial polymer, (*S-A*)_*n*_. The complementary partner of fragment *A*, i.e., fragment *B*, is also conjugated to *S* to render another polymer (*S-B*)_*m*_, where *m* and *n* represent the number of repeating units. In a mixed solution of (*S-A*)_n_ and (*S-B*)_*m*_, the pairwise interactions between *A* and *B* act as inter-strand cross-linkers to generate a cross-linked polymer network. Specifically, the two halves from the native folded small protein GL5, G_N_ and G_C_ fragments, corresponding to the split fragments *A* and *B* mentioned-above, respectively, are capable of reconstituting by domain swapping. Using the G_N_ and G_C_ fragments as sticky nodes, the mixture of two engineered proteins, (I27-G_N_-I27)_4_ and (I27_3_-GC)_3_, in which I27 represents an 89-residue spacer S domain, shows a readily potency to form a transparent solid hydrogel at a protein concentration of 10% mass/volume (Figure 7B). When the solution temperature is above 23°C (the melting temperature of the reconstituted G_N_/G_C_), the hydrogel melts into a viscous solution. Upon cooling, the viscous solution changes to hydrogel reversibly. The reversibility of solution-gel phase transition upon heating demonstrates that the (I27-G_N_-I27)_4_ and (I27_3_-GC)_3_ co-assembled system is consistent with the energetic characteristics of the dynamic conformational landscape of a protein domain swapping (Yang et al., 2004). Moreover, there are two cysteine residues located in G_N_ and G_C_ fragments, respectively, allowing for the formation of a disulfide bond in the reconstitution of GL5 under oxidizing conditions. Thus, the co-assembled hydrogel displays an oxidation-triggered structural conversion from physical cross-linking to chemical cross-linking. The erosion profile of oxidized hydrogels is more stable than that of reduced hydrogels. For instance, reduced hydrogels were completely eroded in 9 days. In contrast, ~50% of the oxidized hydrogels remained intact after the same time course. The transition from physical to chemical cross-linking is reversible by adding reductive species to switch the redox state of the two cysteine residues.

## Applications of Designed Self-Assembling Protein Architectures in Medicine

The artificially designed protein supramolecules exhibit enormous potentials in biological medicine due to their nano-size effect, multi-functional integration, biocompatibility, and stability. Herein, we review the recent progress made by applying self-assembled protein materials in the research fields of vaccine, enzyme, and drug delivery.

**Figure 8:**
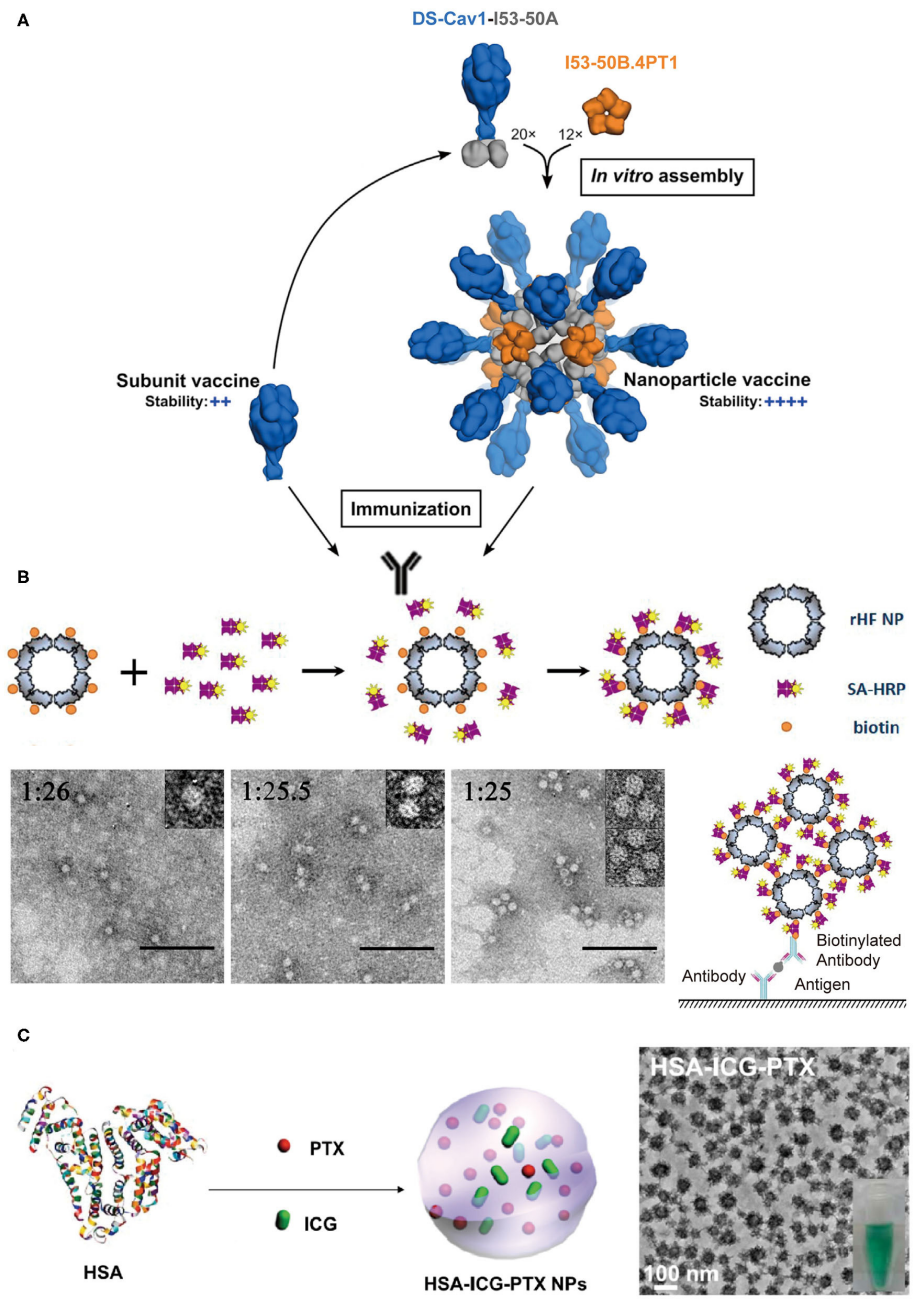
Protein assemblies for medical applications. **(A)** A schematic illustration of the assembly structure of a DS-Cav1 based nanoparticle vaccine. Adapted with permission from Marcandalli et al. (2019). Copyright 2019 Elsevier Inc. **(B)** The molecular mechanism proposed for the co-assembly of ENC (top) and the particle geometry of ENC observed in TEM images with different molar ratios of rHF NP to SA-HRP of 1:26, 1:25.5, and 1:25, respectively (bottom). The scale bar is 100 nm in each TEM images. Reproduced with permission from Men et al. (2015). Copyright 2015 American Chemical Society. **(C)** A schematic illustration of the formation of HSA-ICG-PTX nanoparticles based on the co-assembly of HSA, PTX, and ICG. Adapted with permission from Chen et al. (2015). Copyright 2014 WILEY-VCH.

Vaccines that induce the immune system of a host to produce neutralizing antibodies against the infection of pathogens play a profound role in the prevention of diseases. Conventional vaccines including inactivated or live-attenuated pathogens pose a challenge to the pharmaceutical cold chain logistics (Cruz-Resendiz et al., 2020; Malonis et al., 2020). To facilitate the storage and transportation of vaccines, subunit and recombinant vaccines that consist of peptides or proteins derived from B-cell or T-cell epitopes are created to achieve enhanced stability. However, the reduced antigen size of subunit vaccines greatly weakens their immunogenicity, and thus multiple immunizations are needed to reach an effective immune response (Cruz-Resendiz et al., 2020; Malonis et al., 2020). To overcome this drawback, an altered strategy that uses self-assembled proteins as scaffolds to regularly array multiple antigens on the surface of vaccines is proposed. This approach has two benefits. First, the increased apparent molecular weight of assembled subunit vaccines effectively raises the level of an immune response upon vaccine challenge. Second, a higher local concentration of antigens is achieved to facilitate the multivalent binding interactions with the antigen-binding receptors on the surface of B-cell or T-cell. For example, hemagglutinin and neuraminidase are the envelope proteins anchored on the external surface of the influenza H9N2 virus and function as epitopes to stimulate the host immune system. Matrix protein M1 is a structural protein underneath the viral envelop and interacts with the cytoplasmic tails of hemagglutinin and neuraminidase. The co-expression of these three proteins in an Sf9 insect cell line leads to the secretion of hemagglutinin/neuraminidase/M1 co-assembled virus-like particles (VLPs) (Pushko et al., 2005). The diameter of artificial VLPs was determined to be approximately 80–100 nm, which is close to the size of the native influenza H9N2 virus. VLPs retain the immunogenicity of hemagglutinin and neuraminidase. After a subcutaneous inoculation of VLPs with a BALB/c mice model, the antibodies specific for the influenza H9N2 virus were identified from the serum of mice. The replication of the influenza virus was also inhibited by the VLPs' challenge. The titers of replicating H9N2 virus in lungs were decreased to be <2 log_10_ 50% egg infectious doses per milliliter of the virus, whereas the titers of virus were unmeasurable in noses on the 5th day after an intramuscular administration of VLPs (Pushko et al., 2005; Kang et al., 2009). The assembled protein strategy can be leveraged to the design of vaccines against other types of infectious diseases. Respiratory syncytial virus (RSV) is a leading pathogen that causes lung and respiratory infections, especially in infants and children. Current options for preventing RSV are limited and numerous attempts to develop vaccines against RSV came to naught. Fusion glycoprotein is an envelope protein, mediating the fusion process of the RSV viral and mammalian cell membranes. Marcandalli et al. (2019) used two structural proteins, I53-50A and I53-50B.4PT1 as a scaffold to tether an RSV fusion protein derivative, DS-Cav1, as trimer on the exterior surface of an assembled protein nanoparticle with a surface density of 20 DS-Cav1 trimers per particle (Figure 8A). This assembled protein nanoparticle exhibited an improved immunogenic response to induce neutralizing antibody, which is roughly 10-folds higher than that of free DS-Cav1 trimers.

The spatial organization of proteins with enzyme activity into a hierarchical architecture can lead to a series of beneficial outputs in the efficiency of enzymatic catalysis. (1) The density of active sites is increased by the assembly of enzymes. Enzyme-linked immunosorbent assay (ELISA) is a broadly used analytical method for quantifying the concentration of antigen in a sample, and horseradish peroxidase (HRP) is the key enzyme component for ELISA to magnify the detection signal. Men et al. (2015) used streptavidin-labeled HRP (SA-HRP) to assemble with biotinylated ferritin nanoparticles (bFNP) via a streptavidin-biotin pair and constructed an enzyme nanocomposite (ENC) with a high-density immobilization of HRP. Compared to the free HRP, the ENC-based ELISA assay displays a 10,000-fold increase in the reaction rate of enzymatic catalysis and an improvement in the ELISA sensitivity (Men et al., 2015) (Figure 8B). (2) The supramolecular framework formed by the assembly of enzymes improves the stability of enzymes. For example, a recombinant nano-selenoenzyme SP1-GPx was designed by conjugating two functional moieties: (i) a stable protein one (SP1) that drives protein to assemble to a ring-shaped frame, and (ii) a glutathione peroxidase (GPx) that maintains an active selenocysteine to scavenge free radical (Miao et al., 2014). The self-assembly of SP1-GPx presents an ordered GPx pattern on the SP1 frame surface and changes the enzymatic properties of GPx. Specifically, the catalytic reactivity of nanostructured selenoenzyme (390.6 U μmol^−1^) is higher than the catalytic reactivity of free GPx (302 U μmol^−1^). The thermostability of the enzyme is promoted to allow SP1-GPx to work over a broad temperature range from 20 to 85°C. Given the fact that the physiological temperature of mitochondria, one of the main free radical producers, is close to 50°C, it is reasonable to predict that the SP1-GPx assembly possesses an excellent capability to protect cells from oxidative damage at the mitochondrial level (Chretien et al., 2018). (3) Positive cooperativity between heterogeneous subunits is built via sequencing different types of enzymes into a multi-enzymatic system (Cao et al., 2019). For instance, the menaquinone biosynthetic pathway from chorismate is a complicated multi-step process involving the isomerization of chorismate, the addition reaction of isochorismate with α-ketoglutaric acid, the aromatization of 2-succinyl-6-hydroxy-2,4-cyclohexadiene-1-carboxylic acid, etc. The structural conversion from chorismate to menaquinone is catalyzed by a group of enzymes including MenF, MenD, and MenH (Liu et al., 2019). Liu et al. (2019) used ELP as a building block to achieve two types of protein scaffolds, a cross-linked scaffold via the coupling between different ELP units and a cyclic scaffold via a head-to-tail-cyclization of one ELP unit. Enzymes MenF, MenD, and MenH were assembled on both scaffolds. Relative to the 76% catalytic yield of the free-floating enzymes, the catalytic yield was greatly increased to 94.4% for the cross-linked scaffold group and 93.7% for the cyclic scaffold group (Liu et al., 2019).

Protein assemblies also exhibit a strong potential to serve as drug carriers due to their desirable biocompatibility, versatile molecular structures, and editable *in vitro* and *in vivo* functions in a biological system. The utilization of protein assemblies as delivery systems benefits the conventional molecular chemotherapeutics with several promising properties, such as the improved solubility, elongated residence time in the circulation, and the enhanced permeability and retention (Aluri et al., 2009; Danhier et al., 2010). The nanostructure formed by human serum albumin (HSA), an effective delivery carrier for therapeutic agents, has been used in the clinical practice. For example, the albumin nanoparticle loaded with paclitaxel, i.e., Abraxane®, has been approved for treating several cancers including metastatic breast cancer, non-small cell lung cancer, and advanced pancreatic cancer. The physical adsorption of drugs by HSA nanostructure greatly improves the pharmacokinetic profile, increases the solubility of the drug, and reduces the immune allergic response (Kratz, 2014; Zong et al., 2017). Inspired by Abraxane®, Chen et al. (2015) loaded HSA-assembled nanoparticles with a chemotherapeutic agent paclitaxel and a photothermal agent indocyanine green (ICG) to create a dual functional nanodrug (Figure 8C). Such a therapeutic supramolecular system has been approved by the FDA and will benefit the treatments of subcutaneous and metastatic tumors.

## Applications of Designed Self-Assembling Protein Architectures in material Science

Proteins composed of amino acids with a variety of side chains are the most versatile building blocks for bottom-up construction. Meanwhile, proteins offer enormous diversities in terms of structures that would also convert into amazing functional diversities. To date, proteins as building blocks have shown great potential to construct tailored designed systems, including nanofabrication and generation of novel protein-based biomaterials (Dedeo et al., 2010; Witus and Francis, 2011; Hainline et al., 2019; Ilamaran et al., 2019). Herein, we introduce several versatile platforms based on simple protein building blocks for the construction of multiple functional biomaterials.

### Protein Assemblies for Biomechanical Applications

**Figure 9:**
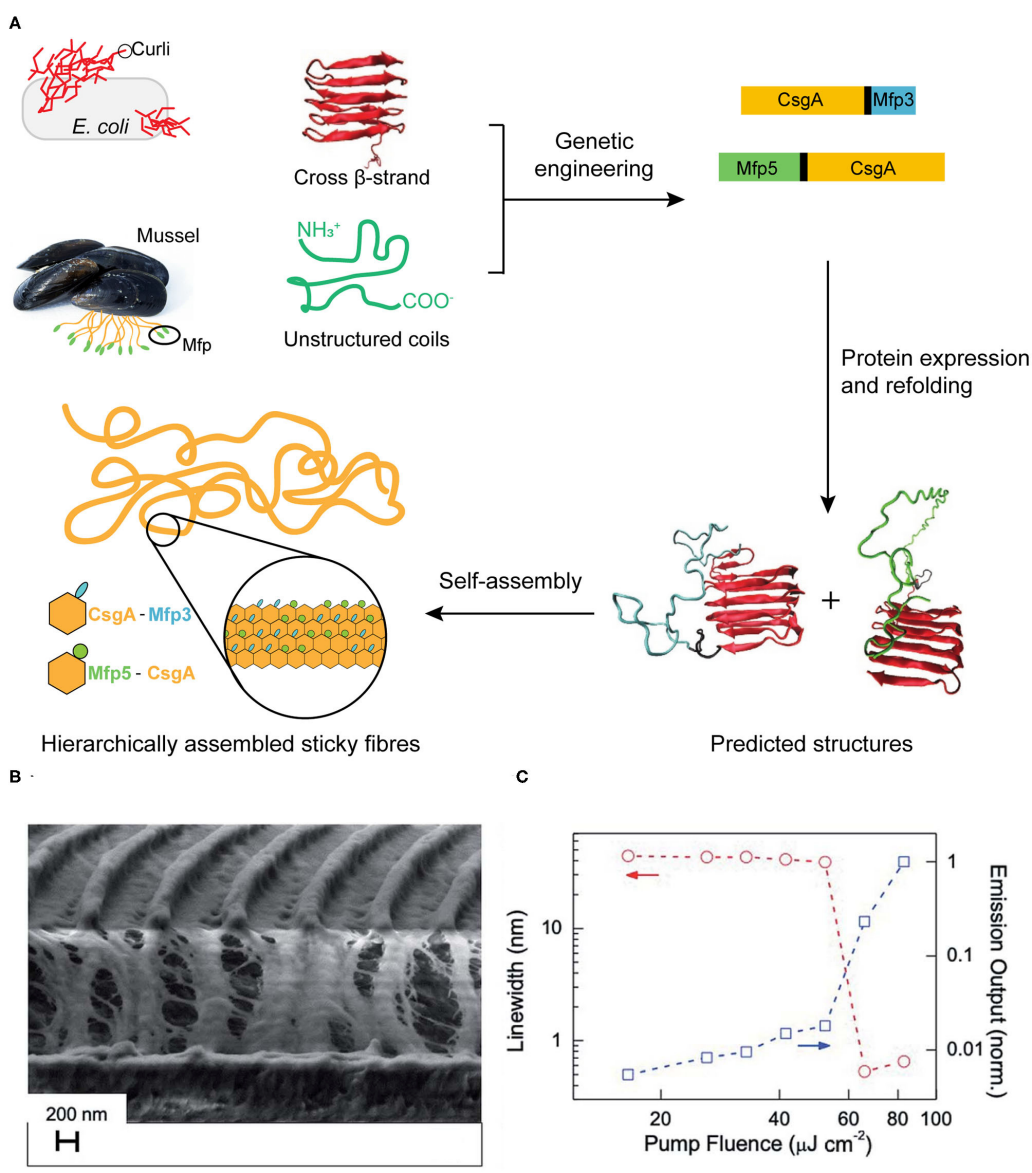
Protein assemblies for bio-mechanical **(A)** and bio-optical applications **(B,C)**. **(A)** Scheme of combinatorial and modular genetic strategy for designing self-assembling underwater adhesives. **(B,C)** Engineered CTPR-based functional nanopatterned materials for bio-optical devices. **(B)** Scanning electron microscope image (vertical cut) of nanostructured CTPR-Rh6G on top of IPS® with 416 nm periodicity. **(C)** Log-log plot of the PL linewidth (left *Y*-axis, circles, indicated by red arrows) and emission output (right *Y*-axis, squares, indicated by blue arrows) normalized by the output at the highest fluence as a function of excitation fluence. Reprinted with permission from Sanchez-deAlcazar et al. (2019). Copyright 2019 Royal Society of Chemistry.

Mussels attach to solid surfaces in the sea, whose adhesives need to be rapid, strong, and tough, otherwise they will be dislodged and dashed by the incoming wave. Similarly, strong underwater adhesives are urgently needed for technological and biomedical applications in water or under the high-moisture environment, such as ship repair and tissue adhesion (Lee et al., 2011; Dolgin, 2013). Two sets of native protein assemblies have been applied in the field of strong underwater adhesives. (1) DOPA (3,4-dihydroxyphenylalanine)-containing biomaterials have been widely investigated for underwater adhesives because of its natural occurrence from marine organisms (Hwang et al., 2004). (2) Amyloidal protein assembly into fibrillar structures have also been exploited to have intrinsic advantages for interfacial underwater adhesion, including self-healing originating from self-polymerization, high tolerance to environmental deterioration, and the large surface areas to enhance adhesion (Chapman et al., 2002; Knowles and Buehler, 2011). Thus, strong underwater adhesives are designed by combining these two independent natural adhesion systems. By using combinatorial and modular genetic strategy, DOPA-based adhesives (Mfp3 and Mfp5, the representatives of DOPA-based mussel adhesives originating from *Mytilus galloprovincialis*) and amyloid-based adhesives (CsgA, the major subunit of adhesive curli fibers in *Escherichia coli*) were constructed (Zhong et al., 2014) (Figure 9A). Molecular dynamics and characterization on the adhesive fibrils showed that CsgA-Mfp3, Mfp5-CsgA, and (CsgA-Mfp3)-co-(Mfp5-CsgA) copolymers of the two fusion proteins construct stable amyloidal structures dominated by the CsgA domains, and the external to the amyloid core is enwound with the highly disordered Mfp domains. The underwater adhesion performance of adhesive fibrils was assessed by using atomic force microscopy (AFM) colloidal probe technique, which essentially measures the asymmetric adhesion mediated by fibrils. The results showed that the fibrils have an underwater adhesion energy of approaching 20.9 mJ/m^2^. This work demonstrates that the amyloidal fibril structures formed by CsgA enable large surface areas for contact, and the multiple disordered Mfp domains on surfaces interact with substrates to achieve an enhanced adhesion performance.

### Protein Assemblies for Bio-optical Applications

Proteins can self-assemble into complex hierarchical nanostructures and also template intricate photoactive, electroactive components, and nanomaterials to fabricate multiple protein-based hybrid functional biomaterials. The protein-based supramolecular structures not only provide ordered control over the arrangement at the nanometer scale, but also confer new properties to photo-/electro-active systems and nanomaterials, such as chirality etc. (Mejias et al., 2016a,b; López-Andarias et al., 2017).

The design and application of protein scaffold based on the pattern formed by engineered protein repeats have been widely investigated, due to their unique advantages of encompassing both the structural simplicity and the intrinsic functional versatility of proteins. A designed consensus tetratricopeptide repeat protein (CTPR) is a representative repeat module, which comprises of 34 amino acid residues and folds into a helix-turn-helix superhelical structure with eight repeats per superhelical turn (D'Andrea and Regan, 2003). There are few conserved residues participating in defining the TPR scaffold structure, which is conducive to manipulating the scaffold while retaining its structural stability. For instance, mutated CTPR protein (M14C, Y17C) can act as precise templates to organize orderly aggregated photoactive porphyrins molecules along the protein structure, while the protein scaffold retains its signature helical structure and assembly properties. This solid thin film with CTPR-porphyrin hybrid imposes order and chirality into the porphyrin arrangement and thus the anisotropic photoconductivity was successfully achieved (Mejias et al., 2016b).

In addition, a potential optical application with nanostructured CTPR films was constructed with an efficient fluorescent dye Rhodamine 6G (Rh6G) (Sanchez-deAlcazar et al., 2019) (Figures 9B,C). The CTPR-Rh6G films exhibit photostability, homogeneity and the emission features that result in the observing light amplification upon photoexcitation with laser pulses. The nanostructured CTPR-Rh6G films were obtained by depositing the protein on an intermediate polymer stamp (IPS®, Obducat) grating templates of 416 nm periodicity. The laser action characterization showed that CTPR–Rh6G films behaved as second order surface emitting distributed feedback (DFB) lasers, emitting sharply (0.5 nm linewidth) centered at 625 nm. Upon changing the excitation density, the onset for laser action showed a sudden change in the slope of the emission output at fluences above a 55 μJ cm^−2^ lasing threshold. This measurement value was below the reported ones in DFB lasers, such as 1.7 mJ cm^−2^ observed on Rh6G-doped silk fibroin or 140 μJ cm^−2^ measured in Rh6G-doped cellulose acetate. This solid-state biological lasing platform opens up possibilities for combining biological media with light sources aiming at *in vivo* imaging or diagnosis, etc.

### Protein Assemblies for Bio-electronic Applications

The application of protein scaffold has also been investigated in the bioelectronic field. For example, CTPR proteins endowed with porphyrin (P) units (photoactive and electron donating), can efficiently wrap around single wall carbon nanotubes (SWCNT) (electron accepting) to form water-processable CTPR-P-SWCNT donor-acceptor bio-nanohybrids (López-Andarias et al., 2017). The photoexcitation of CTPR16-P/CNT by 420 nm laser pulses showed photoconductive nature and presented a remarkable enhancement on the photoconductivity values. The flash-photolysis microwave conductivity (FP-TRMC technique) also demonstrated that the major charge carriers of electrons were injected into the electron accepting SWCNTs and moved along these 1D structures. Briefly, this protein-organic-nanomaterial hybrid demonstrated the photo-absorption capability, excellent conductivity as well as water-based film processability.

**Figure 10:**
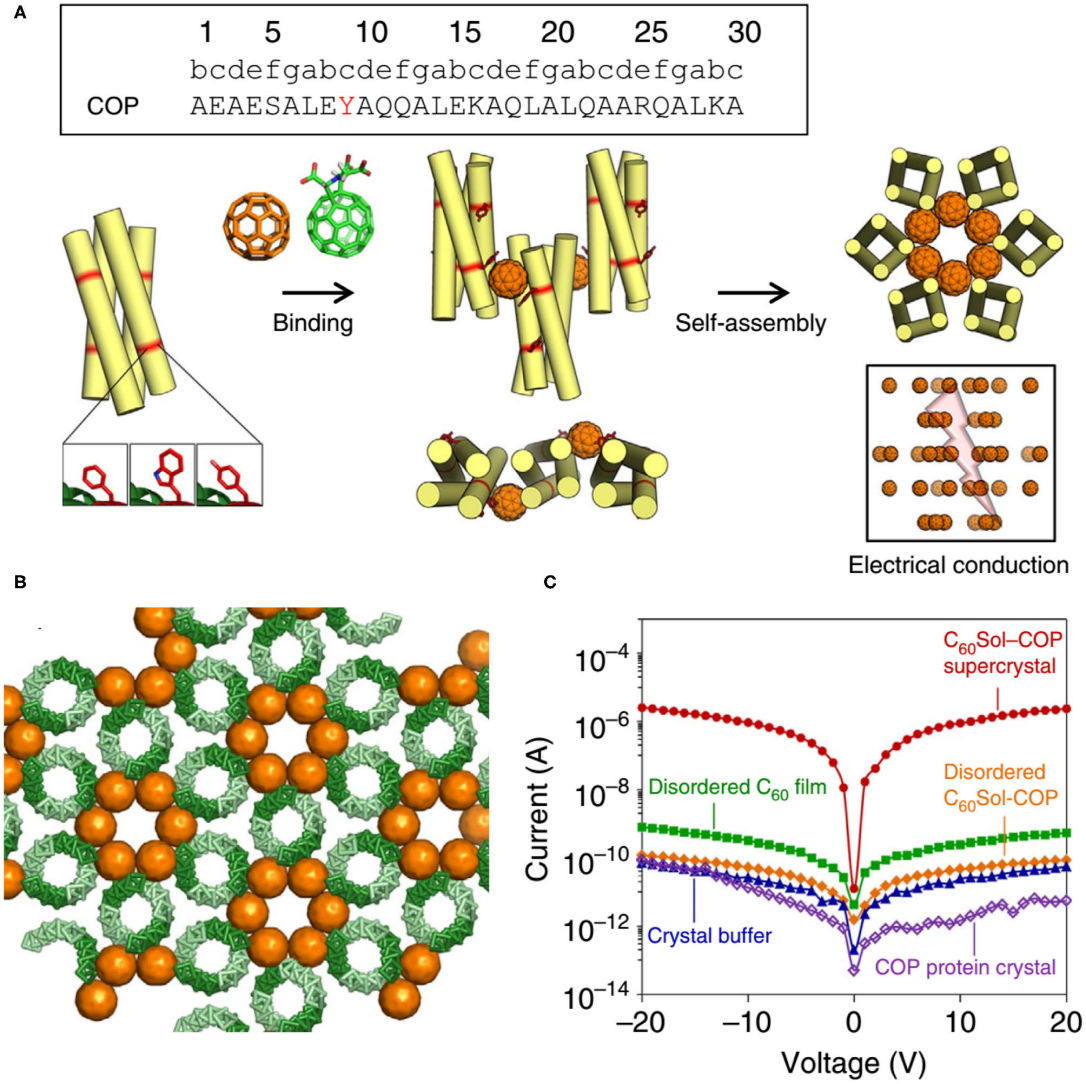
Protein assemblies for bio-electronic applications. **(A)** COP interacts with C_60_ by means of a surface-binding site that includes Tyr residues, and self-assembles into a co-crystalline array with fullerene. **(B)** The C_60_Sol-COP crystal lattice. **(C)** Semi-logarithmic current-voltage characteristic of C_60_Sol-COP super crystal (red dots) and disordered C_60_Sol-COP (orange diamonds) and other controls. Reprinted with permission from Kim et al. (2016). Copyright 2015 Macmillan Publishers Limited.

Here we also introduce an interesting discovery: although the protein-alone crystal and nanomaterial are both electrically insulating, the protein-nanomaterial supercrystals strikingly exhibit high charge conductance (Kim et al., 2016) (Figure 10). The designed protein adopts to a canonical antiparallel coiled-coil tetrameric structure (PDB No. 3S0R) at mM concentrations. This C_60_-organizing peptide (hereafter referred to as COP) exposes four tyrosine residues and engages a C_60_ moiety. X-Ray diffraction characterization of C_60_Sol-COP supercrystals demonstrates that C_60_ groups occupy periodic lattice sites, sandwiched between two Tyr residues from the neighboring tetramers, presenting the helix(Tyr)-C_60_-helix(Tyr)-binding mode. The nm-spaced helical arrangement of C_60_ groups along a crystallographic axis endows the supercrystals with high electrical conductance properties (1.40 × 10^−7^ S, corresponding to a resistance of 7.14 × 10^6^ Ω) with at least four orders of magnitude higher currents than in any of the controls, including COP-alone protein crystal, disordered C_60_Sol-COP, etc. This discovery offers an exciting direction of inquiry and design of novel properties through generating protein-fullerene assemblies.

## Conclusion and Challenges

We have overviewed the recent advances made for the programmable self-assembly of proteins into higher order structures through a *de novo* design of the highly specific biomolecular interactions. Strategies that can be applied to achieve a precise control of hierarchical architectures are summarized and discussed. Taking advantage of the extensive complex architectures formed by protein molecules, diverse well-defined nano-patterns and lattices have been constructed via a bottom-up approach that spans from the single molecular to macroscopic scale. Such nature-inspired supramolecular assemblies demonstrate a tremendous potential in the design and fabrication of innovative nanomaterials and nanodevices with specified functions. Nowadays, the rational design of protein-assembled structure has been emerged as a promoting multidisciplinary research field located at the interface between nanotechnology, synthetic biology, chemical biology, physical chemistry, and biomedical engineering.

This field still has many problems and challenges for the future study. One of the most notorious problems is the robustness of the *de novo* design of protein origami. In contrast to the construction of DNA origami which is robust and readily in practice, the successful design and creation of protein origami is still a small fraction of the total designs attempted (Lapenta et al., 2018). The low controllability of *de novo* designed protein supramolecular structures is correlated to the more complicated pertinent properties of protein relative to DNA (Gradisar and Jerala, 2014). The structural elements of proteins and DNAs are distinct. Protein comprises 20 amino acids with different side chain properties (ionic, non-ionic, polar, and non-polar), whereas DNA contains only four structurally similar nucleotides. The packing of proteins within a protein origami can involve topological patterns of parallel coiled-coil, antiparallel coiled-coil, parallel β-sheet, and antiparallel β-sheet. In contrast, the DNA strands within a DNA origami are typically organized in antiparallel. The intermolecular interactions driving protein folding and assembly include van der Waals interactions, hydrophobic interactions, hydrogen bonds, and electrostatic interactions, whereas the interactions governing a DNA origami are mainly attributed to be the hydrogen bonds between nucleotide base pairs (Gradisar and Jerala, 2014). There remains a vast unexploited field in the understanding of interactions that govern protein folding and association, such as the origins of hydrophobic interactions, and the cooperativity of intermolecular interactions mediated by water at protein surface (Garde, 2015; Ma et al., 2015; Wang et al., 2017). A deep understanding of the physico-chemical roles implemented by 20 amino acids can improve the success rate of protein origami design. The advanced technologies and methodologies which provide information on the changes of protein dynamics and folding conformational landscapes along the reaction pathway, such as modified chemical exchange saturation transfer (CEST) and the Car-Purcell-Meiboom-Gill-based relaxation dispersion experiments using nuclear magnetic resonance spectroscopy, will also help to reveal the underlying mechanism of how these proteins fold and function (Niu et al., 2018; Zhang et al., 2018). Another problem is the fate of artificial protein assembly *in vivo*. When protein assemblies are used as drug carriers or nano-vaccines and injected into the body, enzymes may degrade or modify the integrity, surface chemistry, and the aggregation number of protein assemblies, which further leads to a change in the bio-functionality and bio-distribution (Kreyling et al., 2015; Chen et al., 2017). Two directions are important for the future study. The first will be to track the fate of *de novo* protein assembly *in vivo*. Such measurement is difficult because we lack an appropriate analytical instrument and method for evaluating the stability and structural conversion of protein assemblies *in vivo*. More efforts in this direction are anticipated. The second will be to improve the stability of protein assembly in the medium *in vivo*, which is imperative for the future clinical applications.

Although studies in this field are still in a preliminary stage, the versatile structures and properties of protein assemblies have provided informative instructions for generating biomaterials and nanomaterials. Further development of protein-based supramolecular system and the rational design strategy will guide the progress of protein-assembled materials. Protein assemblies will continue to contribute to the development of material science and elicit multidisciplinary efforts for its further achievements.

## Author Contributions

All authors listed have made a substantial, direct and intellectual contribution to the work, and approved it for publication.

## Conflict of Interest

The authors declare that the research was conducted in the absence of any commercial or financial relationships that could be construed as a potential conflict of interest.
